# Comparison of Drusen Volume Assessed by Two Different OCT Devices

**DOI:** 10.3390/jcm9082657

**Published:** 2020-08-17

**Authors:** Marco Beck, Devika S. Joshi, Lieselotte Berger, Gerd Klose, Sandro De Zanet, Agata Mosinska, Stefanos Apostolopoulos, Andreas Ebneter, Martin S. Zinkernagel, Sebastian Wolf, Marion R. Munk

**Affiliations:** 1 Department of Ophthalmology, Inselspital, Bern University Hospital, University of Bern, 3010 Bern, Switzerland; marco.beck@insel.ch (M.B.); devika.joshi1788@gmail.com (D.S.J.); lieselotteerika.berger@insel.ch (L.B.); andreas.ebneter@insel.ch (A.E.); martin.zinkernagel@insel.ch (M.S.Z.); sebastian.wolf@insel.ch (S.W.); 2 Carl Zeiss Meditec, Inc., Tokyo 102-0083, Japan; gerd.klose@zeiss.com; 3 RetinAI Medical AG, 3010 Bern, Switzerland; sandro@retinai.com (S.D.Z.); agata@retinai.com (A.M.); stefanos@retinai.com (S.A.); 4 Bern Photographic Reading Center, Inselspital, Bern University Hospital, University of Bern, 3010 Bern, Switzerland

**Keywords:** optical coherence tomography, age-related macular degeneration, AMD, drusen, drusen volume, segmentation, retinal pigment epithelium, spectral-domain OCT, swept-source OCT, retinal imaging analysis, segmentation

## Abstract

To compare drusen volume between Heidelberg Spectral Domain (SD-) and Zeiss Swept-Source (SS) PlexElite Optical Coherence Tomography (OCT) determined by manual and automated segmentation methods. Thirty-two eyes of 24 patients with Age-Related Macular Degeneration (AMD) and drusen maculopathy were included. In the central 1 and 3 mm ETDRS circle drusen volumes were calculated and compared. Drusen segmentation was performed using automated manufacturer algorithms of the two OCT devices. Then, the automated segmentation was manually corrected and compared and finally analyzed using customized software. Though on SD-OCT, there was a significant difference of mean drusen volume prior to and after manual correction (mean difference: 0.0188 ± 0.0269 mm^3^, *p* < 0.001, corr. *p* < 0.001, correlation of r = 0.90), there was no difference found on SS-OCT (mean difference: 0.0001 ± 0.0003 mm^3^, *p* = 0.262, corr. *p* = 0.524, r = 1.0). Heidelberg-acquired mean drusen volume after manual correction was significantly different from Zeiss-acquired drusen volume after manual correction (mean difference: 0.1231 ± 0.0371 mm^3^, *p* < 0.001, corr. *p* < 0.001, r = 0.68). Using customized software, the difference of measurements between both devices decreased and correlation among the measurements improved (mean difference: 0.0547 ± 0.0744 mm^3^, *p* = 0.02, corr. *p* = 0.08, r = 0.937). Heidelberg SD-OCT, the Zeiss PlexElite SS-OCT, and customized software all measured significantly different drusen volumes. Therefore, devices/algorithms may not be interchangeable. Third-party customized software helps to minimize differences, which may allow a pooling of data of different devices, e.g., in multicenter trials.

## 1. Introduction

Along with pigment changes, drusen are some of the earliest signs of Age-Related Macular Degeneration (AMD). Drusen are described as focal deposits of extracellular debris between the basal lamina of the Retinal Pigment Epithelium (RPE) and the inner collagenous layer of the Bruch’s membrane [1]. Drusen are an important surrogate for progression of AMD [2] and hence techniques and tools to detect and analyze the area and volume [3] of drusen are important for long term follow-up of such patients. Additionally, quantitative drusen changes have been regularly assessed in trials as secondary or exploratory outcome parameters to prove efficacy of potential treatments and to follow disease progression of AMD [2]. Until now the increase of drusen area and the maximal drusen size were mainly assessed by color fundus photography [4,5]. However, these assessments may vary, as variable reproducibility has been reported in the measured drusen area due to fundus pigmentation, media opacities and quality of photographs [6,7].

Spectral Domain Optical Coherence Tomography (SD-OCT) is nowadays widely used in ophthalmology [8] and inbuilt software allows one to segment individual retinal layers and thus quantify drusen volume [9,10,11]. Additionally, a custom-made software has been developed to quantify drusen on OCT [12,13,14]. These algorithms are dependent on the detection of the surface of the RPE, an estimated fit of the original or expected RPE baseline (assuming no elevation) or the Bruch’s membrane. Although the reproducibility of these algorithms for quantifying drusen has been previously evaluated [15,16], comparisons between such algorithms of different SD-OCT and SS-OCT devices are scarce.

In this report, we compare OCT drusen volume determined by two different OCT devices (Heidelberg Spectralis OCT and Zeiss PlexElite SS-OCT) using manufacturers’ software and a customized, third party segmentation software. We further compare the automatically assessed drusen volume obtained by these machines with that after manual correction of the automated segmentation.

## 2. Experimental Section

Methods: All the investigations followed the ICH-GCP guidelines, which correspond to the Kassebaum–Kennedy Health Insurance Portability and Accountability Act of 1996 (HIPAA) regulations. The institutional review board at the University of Bern, Switzerland approved the study (KEK-2019-01588).

Subjects: Thirty-two eyes of 24 patients from the University Eye Clinic of Bern with significant drusen maculopathy with medium to large sized drusen were included in this retrospective study. Included patients were categorized according to Age-Related Eye Disease Study (AREDS) classification and diagnosed with intermediate or advanced AMD of AREDS category 3 and 4 [17]. They underwent SD-OCT (Spectralis OCT; Heidelberg Engineering, Heidelberg, Germany) and SS-OCT (PLEX Elite 9000; Carl Zeiss Meditec, Inc., Dublin, CA, USA) imaging on the same day covering the central 6 × 6 mm. Only eyes with significant drusen maculopathy showing intermediate and/or large drusen were includes in this study. Eyes with geographic atrophy and/or choroidal neovascularization were excluded.

Imaging protocol: SD-OCT images were performed on a Spectralis OCT, Heidelberg Engineering, Heidelberg, Germany) using 6 × 6 mm macular volume scan consisting of 49 scans (~125 µm spacing between each scan) 9-times averaged, which is the standard OCT protocol at our department and at the Bern Photographic Reading Center (BPRC) for Heidelberg Spectralis OCTs. The same patients/eyes also underwent OCT imaging on the PLEX Elite (PLEX Elite 9000; Carl Zeiss Meditec, Inc., Dublin, CA, USA) using the macular cube protocol (6 × 6 Angio scan, consisting of 500 A scans, 12 µm spacing) centered on the fovea at the same time point. Only volume scans of high image quality were included. For Heidelberg Spectralis OCT a minimum of 20 dB SNR and for Plex Elite OCT a signal strength of ≥5 was required, respectively.

**Figure 1:**
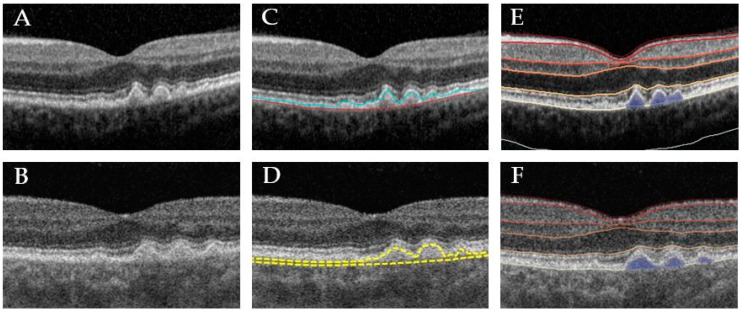
Representative example of different drusen segmentations. Left: unsegmented B-scan of Heidelberg Spectralis Spectral Domain Optical Coherence Tomography (SD-OCT) (**A**) and Zeiss Swept-Source PlexElite Optical Coherence Tomography (SS-OCT) (**B**). Middle: machine inbuilt segmentation of Heidelberg (**C**) and Zeiss SS-OCT ARI network test algorithm based on Cirrus segmentation algorithm (**D**). Right: segmentation of Heidelberg Spectralis SD-OCT (**E**) and Zeiss SS-OCT B-scan (**F**) B-scan using customized, third-party Discovery software, respectively.

Automated and Manual assessment of Drusen volume: Drusen and other RPE elevations such as Pigment Epithelial Detachments (PEDs) were defined as the space between the outer border of the highly reflective RPE band (the outermost of the bright outer bands) and the inner border of the choroid [18]. All visually identifiable drusen were included. A PED was defined as an RPE elevation >250 µm and was included in the drusen volume assessment. Reticular pseudodrusen were not segmented and not included in the analysis. Automated quantification of drusen volume within the central 1 and 3 mm ETDRS circle was performed using the inbuilt Heidelberg segmentation software (Heidelberg Eye Explorer version 1.9.10.0, Heidelberg Engineering, Germany). Respective software delineates 11 different retinal boundaries including the Retinal Pigment Epithelium (RPE), and the Basal Membrane (BM) [9,10] (Figure 1).

The 6 × 6 mm cube scans of the PlexElite were segmented using the ARI Network Test Algorithms Version 0.6.1 (developed by ZEISS Algorithm Development, available at: www.arinetworkhub.com). The algorithm is based on the already approved and commercially available RPE and drusen segmentation algorithm for Zeiss Cirrus [19], though the version used is under development and has not been validated yet. The Advanced RPE Analysis software provides the drusen volume and area of the 3 and 5 mm circles centered on the fovea. The RPE segmentation is measured with reference to the RPE fit line for any kind of RPE elevations. 

The Heidelberg Spectralis automated segmentation algorithm aligns the outer border of the Bruch’s membrane and the RPE (Figure 1). Thus, when drusen volume is assessed with this software, the normal distance between the Bruch’s membrane and the RPE is included. In contrast, the RPE segmentation of Zeiss SS-OCT is measured with reference to the RPE fit line [20]. The automated positioning of the two-segmentation lines is as follows: The RPE line, the inner line follows the RPE, thus the drusen elevations, while the second outer line (so called RPE fit line) is the outer boundary and an approximation of the outer wall curvature. To only assess drusen volume, instead of also including the physiological distance between the RPE and the RPE fit line, this algorithm only considers a distance between the RPE and the RPE fit boundary of >20 µm. So, “real” drusen volume is provided here instead of including the normal distances between respective segmentation lines, which would result in a constant offset in the obtained values.

In a second step, the retinal segmentations of each individual B-scan of both devices were checked and if necessary, manually edited by an experienced retina specialist (DJ). 

Then the segmentations were re-reviewed and if necessary corrected by another independent expert grader (MB and MRM). The drusen values before and after manual correction were recorded and compared. The manually corrected drusen volume was compared between both devices.

**Figure 2:**
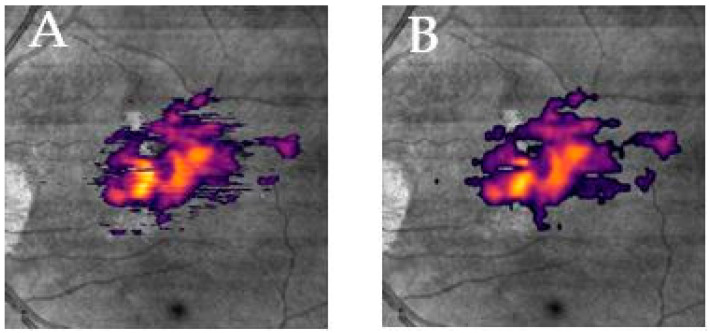
En face view of segmented drusen volume acquired with Zeiss SS-OCT on the infrared image with full segmentation (**A**) and subsampled segmentation (**B**).

In a final step the automated segmentation algorithm of Discovery^®^ (1.3, RetinAI Medical AG, Bern, Switzerland) was employed to compare the drusen volume of the Spectralis images with the PlexElite SS-OCT using a customized, third party, independent segmentation software. The automatic detection method used in Discovery is based on a deep convolutional neural network with encoder–decoder architecture. Each B-scan is processed separately, by segmenting the detachment between the RPE and Bruch’s membrane. Drusen height is computed as height of the detected detachment. The segmentation was checked, but potential segmentation errors were not corrected as no manual correction is possible using the software. To assess the potential impact of the different B-scan spacing of the two devices and protocols on drusen volume, we extracted, in a final step, every 10th scan of the 500 acquired B-scans of the SS-OCT scanning patterns. Thus, we reduced the number of the 500 SS-OCT acquired B-scans to 50 scans with an average distance of 120 µm, which is similar to the Heidelberg SD-OCT scan patterns with acquired 49 B-scans and 125 µm spacing (Figure 2).

Data Analysis: The data was analyzed with SPSS version 23 (IBM, Chicago, IL, USA) using paired T-test and Pearson correlation coefficient. Drusen volume in cubic microns (mm^3^) of the central area of the 1 and 3 mm ETDRS circle before and after manual correction of automated segmentation of both the devices were assessed. The rationale behind assessing the volume of the central 1 and 3 mm ETDRS circle, was that this was the only common measurement provided by both manufacturers’ software. Zeiss software allows the volume assessment of the central 3- and 5-mm circles, while the inbuilt Heidelberg OCT software offers measurements of the 1, 3 and 6 mm ETDRS grid circles. 

The paired T-test was employed to assess intra-device differences of the mean drusen volume before and after manual correction. The T-test compared the central 1 and 3 mm ETDRS circle mean drusen volume of the two different devices using their individual inbuilt software and the customized Discovery software of RetinAI. Last but not least the T-test compared the drusen measurements among the individual manufacturers’ software with the Discovery customized software. 

The Pearson correlation coefficient was used to assess the correlation between automatically assessed and manually corrected drusen volume of each device, the correlation of corrected drusen volume between the two different devices using their inbuilt software and the drusen measurements assessed with the customized software. For all analyses *p* ≤ 0.05 was considered statistically significant; *p*-value correction for multiple testing was done using the Bonferroni correction. 

## 3. Results

### 3.1. Demography

A total of 32 eyes of 24 patients were included in this study (13 women, mean age: 74.8 ± 10). One eye was excluded due to insufficient image quality on both devices According to AREDS classification, 24 eyes (75%) were graded as AREDS category 3 with multiple intermediate drusen (≥ 63 μm) and/or large drusen (≥125 μm). Eight eyes (25%) were classified as AREDS 4. Respective eyes had multiple intermediate drusen and/or large drusen (≥125 μm); and their fellow eyes, which were not included, had either center involving GA (number = 5) and/or CNV (number = 4). Mean measurements for central 1 and 3 mm ETDRS circle drusen volume assessed by the Heidelberg Spectralis SD-OCT and Zeiss Plex Elite SS-OCT are shown in Table 1. 

### 3.2. Intra-Device Comparison before and after Manual Correction

**Figure 3:**
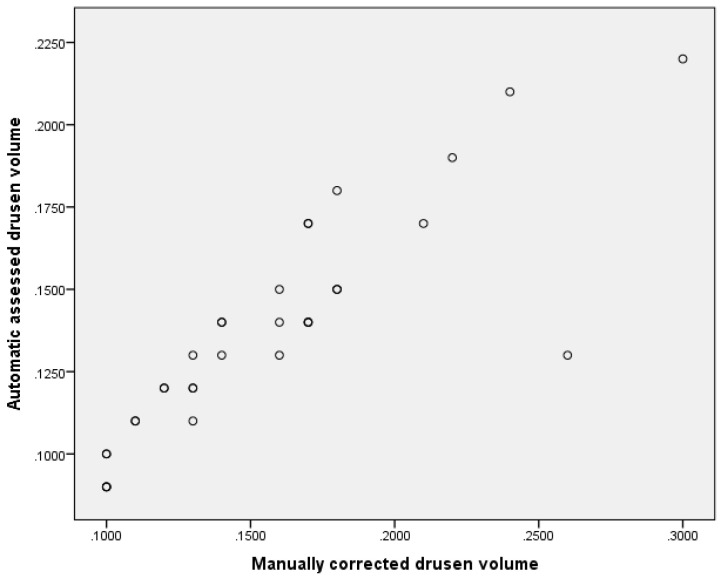
Scatterplot of SD-OCT drusen volume acquired using fully automated segmentation and manually corrected drusen volume. In general, manually corrected drusen volume was larger than automatic acquired volume and discrepancy increased with increasing drusen volume.

There was a significant difference between the mean 1 and 3 mm ETDRS circle drusen volume determined by the Heidelberg SD-OCT manufacturer software prior and after manual correction, respectively (mean difference: 0.0188 ± 0.0269 mm^3^ *p* < 0.001, corr. *p* < 0.001, r = 0.90). A plot is shown in Figure 3. In the Zeiss SS-OCT there was no significant difference between manufacturer automated assessment? mean 1 and 3 mm ETDRS circle drusen volume and manually corrected mean 1 and 3 mm ETDRS circle drusen volume (mean difference: 0.0001 ± 0.0003 mm^3^, *p* = 0.262, corr. *p* = 0.524, r = 1.0). Details are shown in Table 2

### 3.3. Inter-Device Comparison Using Manufacturer Software

**Figure 4:**
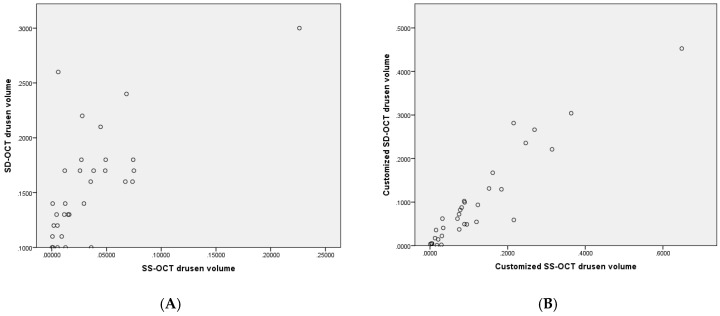
(**A**) Scatter plot illustrating 1 and 3 mm ETDRS circle drusen volume assessed by machine inbuilt algorithm of Heidelberg Spectralis SD-OCT after manual correction (*Y*-axis) vs. the Zeiss SS-OCT assessed and manually corrected 3 mm ETDRS circle drusen volume (*X*-axis). (**B**) Scatter plot illustrating 1 and 3 mm ETDRS circle drusen volume of Heidelberg Spectralis SD-OCT assessed by customized software (*Y*-axis) vs. respective drusen volume of Zeiss SS-OCT assessed by customized software (*X*-axis).

The mean 1 and 3 mm ETDRS circle manually corrected drusen volume determined by Heidelberg SD-OCT was significantly different from the Zeiss SS-OCT assessed and manually corrected 1 and 3 mm ETDRS circle drusen volume with a moderate correlation (mean difference: 0.1231 ± 0.0371 mm^3^, *p*-value ≤ 0.001, corr. *p* ≤ 0.001, correlation of r = 0.681) (Figure 4A). Details are shown in Table 2

### 3.4. Intra-Device Comparison Using Manufacturer vs. Customized Discovery Software

There was a significant difference between the 1 and 3 mm ETDRS circle drusen volumes of the Heidelberg SD-OCT images using the customized discovery software versus the manually corrected manufacturer software (mean difference: 0.0547 ± 0.0744 mm^3^, *p* < 0.001, corr. *p* < 0.001, correlation of r = 0.798). 

Additionally, the Zeiss SS-OCT drusen volume assessed with the customized RetinAI Discovery software significantly differed from the manually corrected Zeiss SS-OCT drusen volume (mean difference: 0.0906 ± 0.101 mm^3^, *p* < 0.001, corr. *p* < 0.001, correlation of r = 0.854). 

The mean SS-OCT 1 and 3 mm ETDRS drusen volume using the customized software did not significantly differ from the subsampled drusen volume (mean difference: 0.0004 ± 0.0063 mm^3^, *p* = 0.733, corr. *p* = 0.733, correlation of r = 0.999). Details are shown in Table 2.

### 3.5. Interdevice Comparison Using Customized Software

Employing the customized software, the difference between the Heidelberg SD-OCT and the Zeiss drusen volume decreased substantially. Consistently, the correlation increased significantly. After correction for multiple testing, the difference was no longer considered statistically significant (mean difference: 0.0547 ± 0.0744 mm^3^, *p* = 0.02, corr. *p* = 0.08, correlation of r = 0.937), (Figure 4B). 

The mean difference decreased further when the SD-OCT drusen volume was compared to the subsampled Zeiss drusen volume (mean difference: 0.0218 ± 0.0514, *p* = 0.023, corr. *p* = 0.069, correlation of r = 0.935). Details are shown in Table 2.

## 4. Discussion

The purpose of this study was to evaluate the reliability and comparability of manual and automated OCT drusen measurements determined by two different devices. 

Comparability of drusen volume among different OCT devices and algorithms are of importance as drusen changes are a hallmark of AMD progression. These changes are tracked in the daily clinical workflow and as surrogate outcome measurements in multicenter trials. 

Drusen ultrastructure can be conveniently imaged with OCT and reliably characterized by readers viewing unprocessed high-resolution scans. Because of the high resolution in SD and SS-OCT scans, the assessment of multiple morphologic parameters of drusen as well as precise characterization of these is possible [21].

In this study, automated as well as manually corrected drusen volume of two different devices and algorithms were compared. In addition, the images of the two devices were compared with an independent customized segmentation software. 

There was a difference between automated acquired SD-OCT drusen volume and manually corrected SD-OCT drusen volume, which implicates that segmentation should be checked prior to interpretation. In particular, in the presence of significant drusen maculopathy, higher the drusen volume, the more likely it is to have segmentation errors and differences between manual corrected and automated acquired values. We show that the drusen volumes acquired with the manufacturer software of both devices are not comparable, and different algorithms led to a significant difference in measurements. This highlights that devices and algorithms are not interchangeable. More so, the data cannot be pooled, which is important for multicenter trials. However, the correlation between both devices was mediocre to strong, which indicates that despite different mean values, there is at least a robust correlation between the measurements. Thus, if one device measures a higher drusen volume the other measures a higher volume, too. 

The different segmentation algorithms are the main reason for the divergent measurements. Applying the same algorithm for OCT segmentation of the two devices, results in better comparability. Drusen volume results acquired with the customized Discovery software revealed a very strong correlation between measurements and the mean differences between both devices substantially decreased below the estimated individual measurements’ precision. This highlights that the utilization of an identical segmentation algorithm is key and can substantially reduce differences. 

While the segmentation lines of the Heidelberg segmentation algorithm are located at the outer border of the Bruch’s membrane and the RPE, which includes the normal distance between the Bruch’s membrane and the RPE, the current Zeiss segmentation borders are following the RPE (and the drusen elevations) and an approximation of the outer wall curvature, the so-called RPE fit line [20]. The Zeiss algorithm further only takes an RPE elevation of >20 micrometers into account, to exclude a nominal distance between Bruch’s membrane and the RPE. Hence, the Zeiss software actually measures “only” the drusen volume, while the Heidelberg software includes the normal volume between RPE and Bruch’s membrane too. The Discovery software in turn does not rely on layer delineation, and rather segments drusen directly. This explains why the measurements differ among the different algorithms. A previous paper by Nittala et al. [22] compared the drusen measurements of the fully automated Zeiss Cirrus 6.0 software, a customized software and a fully manual assessment and revealed that the full form (Intraclass correlation [ICC]) of the measured drusen area between the fully manual versus the fully automated method was lower than the ICC of the drusen volume [22]. The authors assumed that the source of discrepancy was the positioning of the RPE fit line, which was either riding above or below the actual (as determined by the grader) Bruch’s membrane and concluded that the drusen volume was a more robust parameter than the drusen area and should be the preferred metrics for drusen quantification on OCT [23]. As drusen may have poorly demarcated edges, area measurements are likely to be inherently unstable and a small difference in the segmented diameter of a druse can lead to a large change in its area. Due to the topographic profile of the drusen, with small thickness at their edge relative to the center, a small difference in the diameter or border of the drusen will have minimal effects on the volume of large drusen [11]. 

The different B-scan spacing may be another important factor. The 6 × 6 mm SS-OCT consisted of a dense cube, with 12 µm spacing between the individual B-scans, while the SD-OCT scan protocol consisted of 49 individual B-scans with 125 µm spacing. Thus, the interpolation between each scan has a larger impact in a broader scanning protocol compared to a dense scan protocol. While previous studies showed that for healthy eyes, a very dense scan protocol may not be necessary and broader scan patterns provide comparable retinal thickness results as compared to dense scan patterns, one may think that this is not true for diseased eyes and in particular for eyes with drusen, as interpolation may have significant impact on the measurements [24]. One may assume that the broad spacing between the individual B-scans makes it likely that a scan does not cross a druse right through the center, which may have an impact on the volume assessment when the algorithm uses interpolation between the individual B-scans. In addition, a spacing of 125 µm may miss drusen up to 100 µm. Considering the isotropic and dense volumetric sampling of the SS-OCT scan protocol, drusen of all sizes will be detected. On comparison between the two scan patterns, which use the approved advanced RPE analysis software for drusen analysis, and on which the Plex elite SS-OCT drusen assessment was based, the 200 × 200 cube scan pattern, results in an approximate spacing of 30 µm, which is isotropic but drusen smaller than 15 µm will be probably missed. But such pixelwise elevations would anyhow be rather considered ‘noise’ and would be eliminated by the set threshold of >20 µm. Hence, we would not expect significant differences of drusen volume assessment between Plex Elite and the Cirrus 200 × 200 scan pattern [19,25,26]. The Cirrus 512 × 128 scan pattern has a spacing of around 50 µm, i.e., anisotropic and could potentially miss drusen smaller than 35 µm. Presumably, the volume calculation makes some interpolation or assumption, which is not 100% accurate. However, these differences will probably not be clinically relevant.

The Spectralis SD-OCT used a 49-Line scan pattern with a 125 µm spacing, i.e., anisotropic. Drusen up to 100 µm in one direction might not be detected. This indicates that the calculation of a volumetric number, is likely not to be 100% accurate and indicates that using this broad scan pattern on Spectralis will probably lead to an underestimation of the drusen volume as compared to the denser scan pattern of the Zeiss SS-OCT despite of ~20 µm Drusen elevation threshold.

Thus, denser scan patterns provide more accurate measurements than a broader scan pattern for the assessment of drusen volume [27,28]. However, in our study the results did not change when the spacing of 12 µm of the dense Zeiss scan pattern was adapted to a 125 µm spacing, similar to the Heidelberg scan pattern spacing. This is in line with previous papers on the impact of scan densities on central retinal thickness and retinal volume. A broader scan pattern was nearly as accurate as a dense scan pattern. One may note here that included eyes had significant drusen maculopathy with multiple medium and large sized drusen. Thus, small sized drusen which may have been missed on broader scan patterns, probably did not impact drusen volume. Potential differences in eyes with only small sized drusen should be analyzed in future studies to address this question. Although the different scan patterns may be deemed as a limitation of the study, respective protocols and scans were intentionally chosen, as they resemble commonly used scan protocols for respective devices in clinic and in clinical trials. 

In summary, there is high variation in the drusen volume measurements between the two devices, which can be substantially reduced when customized software is used. Measurements of devices are not interchangeable to study progression or regression of drusen volume in a patient. In daily clinic it is therefore beneficial to examine a patient continuously with one device. However, deploying the same algorithm via customized software leads to a substantial decrease of difference in drusen volume and a very strong correlation and may be a valuable tool to allow pooling of data in multicenter trials. Interestingly, scan spacing did not substantially affect the measurements and confirms with previous studies that a broad scan pattern may be as accurate as a dense scan pattern, also for the assessment of drusen volume, at least in eyes with large drusen.

It must be emphasized, however, that the customized software does not fully eliminate differences. Further, this is a small pilot study and to draw clinically meaningful conclusions, larger studies will be needed including more patients, different scan patterns and more devices. Although the Plex Elite algorithm and the customized software algorithm are currently under validation, respective software is not finally validated and commercially available. Moreover, manual correction was performed by a single masked grader and was re-reviewed by another grader. Thus, no masked inter grader reliability for manual correction can be provided, which may be another limitation of this study. 

## Figures and Tables

**Table 1:** Mean measurements for central 1 and 3 mm ETDRS circle drusen volume.

Segmentation	Heidelberg Spectralis SD-OCT(Mean ± SD, mm^3^)	Zeiss Plex Elite SS-OCT(Mean ± SD, mm^3^)
Automatic ^1^	0.1375 ± 0.0329	0.0331 ± 0.0429
Manually ^2^ corrected	0.1563 ± 0.0490	0.0331 ± 0.0428
Discovery ^3^	0.1025 ± 0.1074	0.1237 ± 0.1353
Discovery subsampled ^4^		0.1233 ± 0.1348

^1^ Automatic = automated manufacturer software, ^2^ manually = manually corrected manufacturer segmentation, ^3^ discovery = assessment of drusen volume using customized software, ^4^ Discovery subsampled: Every 10th B-scan was extracted resulting in similar B-scan interspacing of 120 micrometers.

**Table 2:** Inter-device comparison between SD-OCT and SS-OCT device.

Segmentation	Mean difference ± SD, mm^3^	*p*-Value (T-Test)	Correlation (r)
SD Automatic ^1^ vs. SS Automatic ^1^	0.1044 ± 0.0289	*p* < 0.001 corr. *p* < 0.001	0.718
SD Manually ^2^ vs. SS Manually ^2^	0.1231 ± 0.0371	*p* < 0.001 corr. *p* < 0.001	0.681
SD Manually ^2^ vs. SD discovery ^3^	0.0547 ± 0.0744	*p* < 0.001 corr. *p* < 0.001	0.798
SS Manually ^2^ vs. SS discovery ^3^	0.0906 ± 0.101	*p* < 0.001 corr. *p* < 0.001	0.854
SS discovery ^3^ vs. SS discovery ^3^ subsampled ^4^	0.0004 ± 0.0063	*p* = 0.733 corr. *p* = 0.733	0.999
SD discovery ^3^ vs. SS discovery ^3^	0.0547 ± 0.0744	*p* = 0.02 corr. *p* = 0.08	0.937
SD discovery ^3^ vs. SS- discovery ^3^ subsampled ^4^	0.0218 ± 0.0514	*p* = 0.023 corr. *p* = 0.069	0.935

^1^ Automatic = automated manufacturer software, ^2^ manually = manually corrected manufacturer segmentation, ^3^ discovery = assessment of drusen volume using customized software, ^4^ subsampled = every 10th scan was extracted and segmented for drusen volume assessment.
